# Antepartum sleep quality, mental status, and postpartum depressive symptoms: a mediation analysis

**DOI:** 10.1186/s12888-022-04164-y

**Published:** 2022-08-02

**Authors:** Yu Wang, Han Liu, Chen Zhang, Cheng Li, Jing-Jing Xu, Chen-Chi Duan, Lei Chen, Zhi-Wei Liu, Li Jin, Xian-Hua Lin, Chen-Jie Zhang, Han-Qiu Zhang, Jia-Le Yu, Tao Li, Cindy-Lee Dennis, Hong Li, Yan-Ting Wu

**Affiliations:** 1 School of Medicine, International Peace Maternity and Child Health Hospital, Shanghai Jiao Tong University, 910, Hengshan Rd., Shanghai, 200030 China; 2 Shanghai Key Laboratory of Embryo Original Diseases, Shanghai, 200030 China; 3 Obstetrics and Gynecology Hospital, Institute of Reproduction and Development, Fudan University, Shanghai, 200011 China; 4 Bloomberg Faculty of Nursing, University of Toronto, Toronto, Canada; 5 Research Units of Embryo Original Diseases, Chinese Academy of Medical Sciences (No. 2019RU056), Shanghai, China

**Keywords:** Sleep quality, Mental status, Perinatal depressive symptoms, Mediating effect

## Abstract

**Background:**

Poor sleep quality and maternal mood disturbances are common during pregnancy and may play pivotal roles in the development of postpartum depression. We aim to examine the trajectories of sleep quality and mental health in women from early pregnancy to delivery and explore the mediating effects of sleep quality and mental status on the link between antepartum depressive symptoms and postpartum depressive symptoms.

**Methods:**

In an ongoing prospective birth cohort, 1301 women completed questionnaires in the first, second and third trimesters and at 6 weeks postpartum. In each trimester, sleep quality was measured utilizing the Pittsburgh Sleep Quality Index (PSQI), and mental health was assessed with the Center for Epidemiologic Studies Depression Scale (CES-D), the Self-Rating Anxiety Scale (SAS) and the Perceived Stress Scale (PSS). Postpartum depressive symptoms were evaluated by the Edinburgh Postnatal Depression Scale (EPDS). The bootstrap method was used to test the mediation effect.

**Results:**

The PSQI, CES-D, and SAS scores presented U-shaped curves across the antenatal period while the PSS score followed a descending trend. Antenatal sleep quality, depressive symptoms, anxiety symptoms and perceived stress all predicted depressive symptoms at 6 weeks postpartum. The influence of antepartum depressive symptoms on postpartum depressive symptoms was mediated by antepartum sleep quality and anxiety symptoms, which accounted for 32.14%, 39.25% and 31.25% in the first, second and third trimesters (*P* = 0.002, *P* = 0.001, *P* = 0.001, respectively).

**Conclusions:**

Poor sleep quality and anxiety symptoms in pregnancy mediated the relationship between antepartum depressive symptoms and postpartum depressive symptoms. Interventions aimed at detecting and managing sleep quality and elevated anxiety among depressed women in pregnancy warrant further investigation as preventative strategies for postpartum depression.

**Supplementary Information:**

The online version contains supplementary material available at 10.1186/s12888-022-04164-y.

## Background

Sleep, as an essential behavior enabling physical and mental restoration, is associated with multiple health outcomes in the general population. However, women during pregnancy usually confront extraordinary challenges in maintaining good sleep quality with almost half experiencing clinically relevant sleep disturbances [1]. Antenatally, women often complain about reduced sleep duration, poorer sleep quality and more daytime napping, that starts in early pregnancy and significantly worsens by late pregnancy [2, 3]. Hormonal changes during pregnancy can substantially affect sleep architecture [4, 5] as can the physical demands of pregnancy including the growing fetus which adds pressure on the maternal lungs and bladder, affecting maternal normal function, including breathing, urination frequency, and overall comfort while sleeping. This often leads to decreased physical activity levels which further impacts sleep behaviors and mental health. There are numerous pregnancy outcomes in relation to sleep during the perinatal period. In a systematic review of 120 studies incorporating a large scale of pregnant women, sleep disturbances were significantly associated with adverse pregnancy outcomes [6]. Maternal sleep disorders have also been related to changes in interpersonal relationships, difficulties of restoring pre-pregnancy body weight and even child development [7–12]. Recognition of the significance of perinatal sleep on maternal and child health is crucial to envision and meet the needs of women at this critical period.

As with sleep disturbances, many women in pregnancy experience mental health disturbances including depressive symptoms, anxiety symptoms, and elevated stress [13]. Importantly, sleeping problems can take on roles as both a symptom and a causal contributor to poor mental health in pregnancy. A previous study investigating 160 pregnant women found that women with sleep deficiency had higher rates of depressive symptoms across time [14]. In a prospective study examining 257 women, poor sleep quality in early pregnancy could forecast depressive symptoms in late pregnancy [15]. Sleep disturbance is also related to postpartum mental health [16, 17]. Not surprising, a recent review examined the strength of association between antenatal sleep and mental health and demonstrated poor sleep quality and negative psychological state during pregnancy impair maternal and fetal outcomes [18]. Despite the increased attention examining the relationship between sleep disturbances and maternal mental health, there are few studies covering the whole perinatal period from early pregnancy to after delivery. The main purpose of our study is to use mediation analysis to explore the link between sleep quality and mental health during pregnancy coupled with its association with postpartum depressive symptoms.

## Methods

### Participants

This study is a part of the ongoing China National Birth Cohort (CNBC) at the International Peace Maternity and Child Health Hospital in China. The participant inclusion criteria were as follows: 1) age of 18 years or older; 2) expected to search for perinatal care and delivery at the hospital; 3) conceived spontaneously; 4) less than 14 gestational weeks; and 5) planned to live in Shanghai for more than 3 years. Ethics approval was given by the Ethics Committees of the International Peace Maternity and Child Health Hospital (GKLW2016-21), and written informed consent was provided by the participants. All methods were performed in accordance with the relevant guidelines and regulations. Following informed consent procedures, all eligible women completed structured questionnaires before 14 weeks gestation, at 22–26 weeks, 30–34 weeks and 6 weeks postnatally. Women recruited from March 2017 to November 2020 were included in this study. Pregnant women with miscarriages or stillbirths were not included in the further analyses.

### Measurements

#### Sleep quality

Sleep quality was assessed in each trimester with the Pittsburgh Sleep Quality Index (PSQI) [19], a 19-item self-report questionnaire that examines seven dimensions. The PSQI has been psychometrically assessed in Chinese population [20]. The Chinese version of the PSQI has been confirmed with adequate reliability (Cronbach’s α = 0.77–0.84) [21], and has been widely used in pregnant women [12, 22]. In this study a cut-off score of > 5 was used.

#### Depressive symptoms

Depressive symptoms were evaluated in each trimester by the Center for Epidemiologic Studies Depression Scale (CES-D) [23], a 20-item measure with convincing reliability in China (Cronbach’s alpha = 0.82–0.90) [24]. In this study a cut off score ≥ 16 was used. Postnatally, depressive symptoms were evaluated by the Edinburgh Postnatal Depression Scale (EPDS) [25], a 10-item measure that assesses feelings in the last 7 days. The Chinese edition of the EPDS has been verified to have satisfactory reliability and validity (Cronbach’s alpha = 0.87) [26]. In this study, a cutoff score ≥ 10 was used to analyze group differences as this lower cutoff score is suitable for Asian populations [27].

#### Anxiety symptoms

Anxiety symptoms in each trimester was rated on the Zung Self-Rating Anxiety Scale (SAS) [28], a 20-item self-report scale that covers a variety of anxiety symptoms, both psychological and somatic. The Chinese translation of the survey is broadly employed in China, and its validity has been previously reported (Cronbach’s alpha = 0.85) [29]. In this study a cut-off score of ≥ 50 was used.

#### Perceived stress

Perceived stress was measured utilizing the Perceived Stress Scale (PSS) [30], a self-report questionnaire consisting of 10 items that was designed to measure general stress. The Chinese version of PSS has reliable validity in Chinese population (Cronbach’s alpha = 0.85–0.86) [31].

#### Covariates

Maternal age, height, weight, ethnicity, current smoking status, current alcohol drinking status, employment status, educational level and household income were self-reported at baseline in early pregnancy. Accordingly, pre-pregnancy body mass index (BMI) (kg/m^2^) was figured out. Before labor, participants’ weight would be measured and gestational weight gain (GWG) was then computed and classified [32]. Exercise status during pregnancy was recorded at each visit across trimesters. It was evaluated according to the frequency of participation in physical exercise (never, less than once a week and more than once a week). Pregnancy and neonatal outcomes were all obtained from the hospital electronic medical records.

### Statistical analyses

The normality of the data distribution was examined by the Kolmogorov–Smirnov normality test. The characteristics of the pregnant women and newborns were described using median and interquartile range (IQR) for continuous variables and frequencies or proportions for categorical variables. Participants were divided into two groups depending on whether they developed postpartum depressive symptoms (EPDS < 10 vs. EPDS ≥ 10). Differences in maternal characteristics, trimester-specific sleep quality, psychological health, exercise status, pregnancy outcomes and newborn outcomes between the two groups were compared using Mann Whitney U test, Wilcoxon tests, chi-square tests or Fisher’s exact tests when applicable. Variables with a statistical significance of lower than 0.1 were included in further multivariate logistic regression models. The effects of trimester-specific sleep quality and mental status on the postpartum depressive symptoms were figured out using mixed-effects models. All the statistical analyses above were performed with SAS 9.4 and SPSS 25.0. The mediation model was constructed by SPSS 25.0 and AMOS 26.0; the mediation effect was conducted by the bootstrap method. An α-level of 0.05 was used for each analysis.

## Results

### Sample characteristics

In total, 1331 women completed the first trimester questionnaire of which 1327 completed the second trimester questionnaire decreasing slightly to 1314 for the third trimester assessment and the final 6-week postpartum depression measure. Overall, 1301 (97.75%) participants completed all four assessments. Among 1301 participants, there were 39.51%, 28.82% and 37.20% of women had poor sleep quality (PSQI > 5) in the first, second and third trimesters respectively. Correspondingly, the rates of depressive symptoms (CES-D ≥ 16) were 67.02%, 44.39% and 46.96% in the first, second and third trimesters, while the rates of anxiety symptoms (SAS ≥ 50) were 22.92%, 13.87% and 18.11%. Overall, 24.37% (*n* = 317) of women had depressive symptoms at 6 weeks postpartum (EPDS ≥ 10).

When participants were separated into two groups based on whether developing depressive symptoms at 6 weeks postpartum, no differences were found between the two groups on any demographic characteristics. We also evaluated the exercise status in each trimester and found women in postpartum depressed group had less frequency of exercise in the third trimester (*P* < 0.001) (Table 1).

**Table1:** Baseline characteristics of the participants between the two groups in the cohort study

Characteristics	EPDS < 10 (*n* = 984) No. (%)	EPDS ≥ 10 (*n* = 317) No. (%)	*P* value
**Age (years)**			0.080
< 35	770 (78.25)	233 (73.50)	
≥ 35	214 (21.75)	84 (26.50)	
**Pre-pregnancy BMI (kg/m**^**2**^**)**			0.777
< 24	819 (83.23)	266 (83.91)	
≥ 24	165 (16.77)	51 (16.09)	
**Gestational weight gain**			0.348
< optimal	235 (23.88)	66 (20.82)	
optimal	369 (37.50)	132 (41.64)	
> optimal	380 (38.62)	119 (37.54)	
**Ethnicity**			0.161
Han	977 (99.29)	312 (98.42)	
Other	7 (0.71)	5 (1.58)	
**Smoking**			1.000
No	981 (99.70)	317 (100.00)	
Yes	3 (0.30)	0 (0.00)	
**Alcohol drinking**			1.000
No	979 (99.49)	315 (99.37)	
Yes	5 (0.51)	2 (0.63)	
**Employment status**			0.069
Employed	944 (95.93)	311 (98.11)	
Unemployed	40 (4.07)	6 (1.89)	
**Educational level**			0.057
Senior high school or lower	58 (5.89)	10 (3.15)	
Beyond senior high school	926 (94.11)	307 (96.85)	
**Annual household income (CNY)**			0.383
< 200,000	366 (37.20)	106 (33.44)	
200,000–300,000	297 (30.18)	107 (33.75)	
> 300,000	321 (32.62)	104 (32.81)	
**Exercise status in the 1**^**st**^** trimester**			
Never	384 (39.02)	119 (37.54)	0.893
Less than once a week	362 (36.79)	119 (37.54)	
More than once a week	238 (24.19)	79 (24.92)	
**Exercise status in the 2**^**nd**^** trimester**			
Never	450 (45.73)	145 (45.74)	0.809
Less than once a week	175 (17.78)	61 (19.24)	
More than once a week	359 (36.48)	111 (35.02)	
**Exercise status in the 3**^**rd**^** trimester**			
Never	383 (38.92)	123 (38.80)	< 0.001
Less than once a week	154 (15.65)	78 (24.61)	
More than once a week	447 (45.43)	116 (36.59)	

*EPDS* Edinburgh Postnatal Depression Scale, *BMI* Body mass index

### Association between antepartum sleep quality or mental health and postpartum depressive symptoms

**Fig.1:**
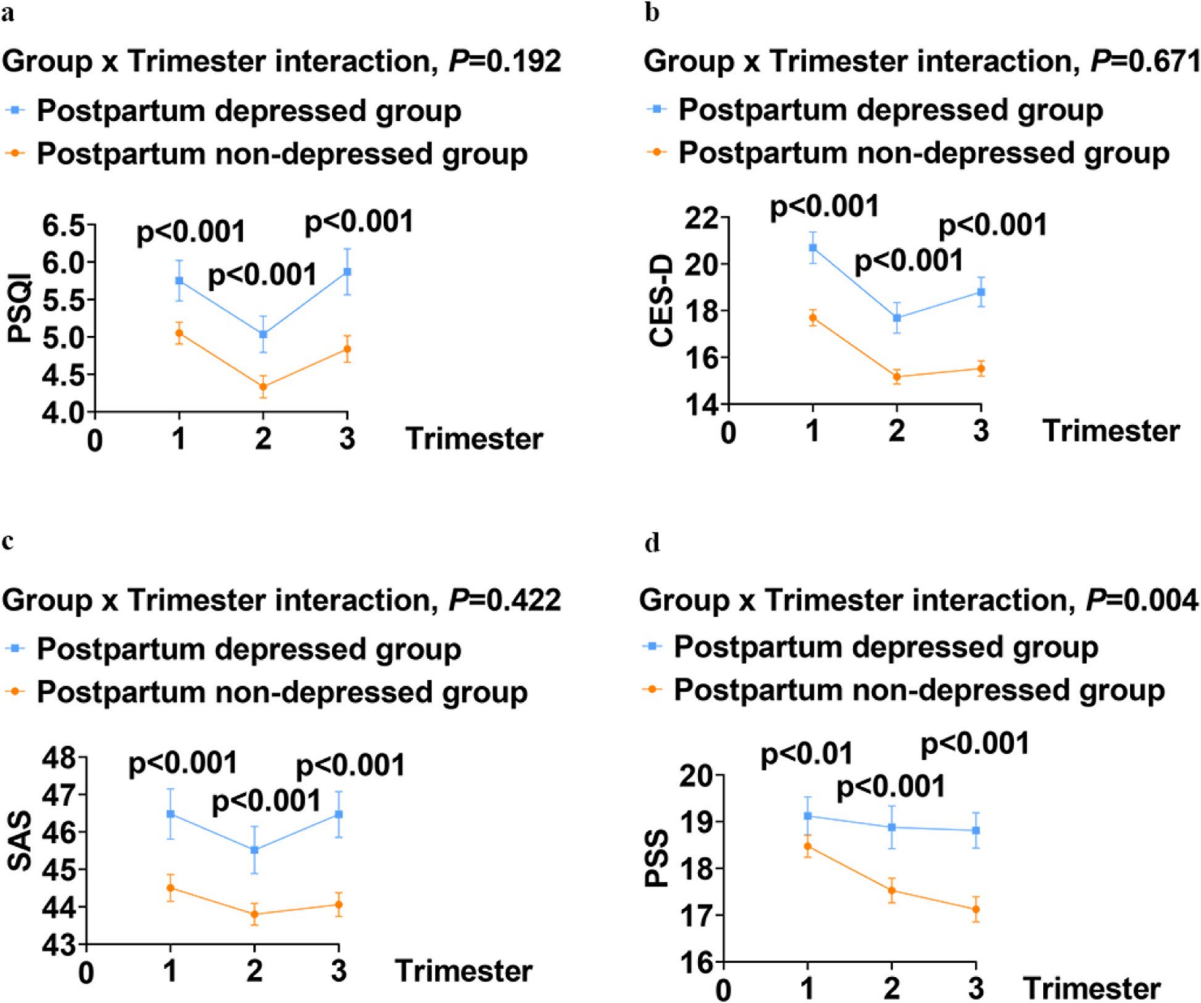
Comparison of psychological health of the participants during pregnancy between the two groups. **a** Sleep quality of participants during pregnancy between the two groups. **b** Depressive symptoms of participants during pregnancy between the two groups. **c** Anxiety symptoms of participants during pregnancy between the two groups. **d** Perceived stress of participants during pregnancy between the two groups

In each trimester sleep quality and mental health symptoms differed significantly between those who developed postpartum depressive symptoms and those who did not. Those with an EPDS ≥ 10 at 6 weeks postpartum had higher PSQI scores and higher rates of poor sleep quality in the first (*P* < 0.001), second (*P* < 0.001), and third (*P* < 0.001) trimesters. Similarly, those with an EPDS ≥ 10 postnatally had higher CES-D, SAS and PSS scores across trimesters. They were more likely to have depressive symptoms, anxiety symptoms and higher level of perceived stress in the first, second, and third trimesters (all *P* < 0.001) (Fig. 1).

Correspondingly, those with poor sleep quality in the first trimester were significantly more likely to have depressive symptoms in the first (*P* < 0.001), second (*P* < 0.001) and third (*P* < 0.001) trimesters and at 6 weeks postpartum (*P* < 0.001). In addition, these women were more likely to have anxiety symptoms in the first (*P* < 0.001), second (*P* < 0.001), and third (*P* < 0.001) trimesters and higher level of perceived stress in the first (*P* < 0.001), second (*P* < 0.001), and third (*P* < 0.001) trimesters (Supplementary Table 1).

Furthermore, we explored obstetric conditions and neonatal outcomes between the two groups and no significant differences were found except for gestational diabetes mellitus (GDM) (*P* = 0.024). Women who had postpartum depressive symptoms were less likely to suffer from GDM (Supplementary Table 2).

Subsequently, the associations between antenatal sleep quality and mental health and postpartum depressive symptoms were explored by multiple logistic regression analysis. PSQI, CES-D, SAS and PSS scores were treated as continuous variables in Table 2. Antenatal sleep quality, depressive symptoms and anxiety symptoms were treated as categorical variables in Supplementary Table 3. In each trimester, higher PSQI, CES-D, SAS and PSS scores indicated higher risk of postpartum depressive symptoms at 6 weeks after adjusting for covariates including age, employment status, educational level, exercise status and GDM. Similarly, women with poor antenatal sleep quality, depressive symptoms and anxiety symptoms were more likely to develop postpartum depressive symptoms.

**Table 2:** Antenatal sleep quality and mental distress predict the risk of postpartum depressive symptoms

	**PSQI** **[OR (95% CI)]**	**CES-D** **[OR (95% CI)]**	**SAS** **[OR (95% CI)]**	**PSS** **[OR (95% CI)]**
**1**^**st**^** trimester**				
Crude	1.13 (1.07, 1.19)	1.09 (1.07, 1.12)	1.06 (1.04, 1.09)	1.05 (1.01, 1.09)
Adjusted^a^	1.13 (1.07, 1.19)	1.09 (1.07, 1.12)	1.06 (1.04, 1.09)	1.04 (1.01, 1.08)
**2**^**nd**^** trimester**				
Crude	1.13 (1.07, 1.19)	1.09 (1.07, 1.12)	1.07 (1.05, 1.10)	1.09 (1.05, 1.13)
Adjusted^a^	1.13 (1.08, 1.20)	1.09 (1.07, 1.12)	1.07 (1.05, 1.10)	1.09 (1.05, 1.12)
**3**^**rd**^** trimester**				
Crude	1.13 (1.08, 1.18)	1.11 (1.09, 1.14)	1.10 (1.07, 1.13)	1.12 (1.08, 1.17)
Adjusted^b^	1.13 (1.08, 1.18)	1.11 (1.08, 1.14)	1.10 (1.07, 1.12)	1.12 (1.08, 1.16)

The PSQI, CES-D, SAS and PSS scores are treated as continuous variables

*PSQI* Pittsburgh Sleep Quality Index, *CES-D* Center for Epidemiologic Studies Depression Scale, *SAS* Self-Rating Anxiety Scale, *PSS* Perceived Stress Scale, *OR* Odds ratio, *CI* Confidential interval

^a^ ORs and 95% CIs were adjusted for age, employment status, education level and exercise status

^b^ ORs and 95% CIs were adjusted for age, employment status, education level, exercise status and gestational diabetes mellitus

Figure 1 presented the trajectories of antenatal sleep quality and mental health in the two groups. The PSQI, CES-D, SAS and PSS scores were all higher in the postnatally depressed group. Additionally, we found that the PSQI, CES-D, and SAS scores presented U-shaped curves across the antepartum period, while the PSS score followed a descending trend in both groups. Using the mixed-effects models, we found the differences in these scores by group were statistically significant (*P* < 0.001), but the interaction effect of group × trimester on sleep quality, mental symptoms presented no significant differences except for perceived stress (*P* = 0.004) after adjusting for covariates such as age, employment status, educational level, exercise status and GDM.

### Mediating effect of antepartum sleep quality and anxiety symptoms in antepartum depressive symptoms and postpartum depressive symptoms

**Fig. 2:**
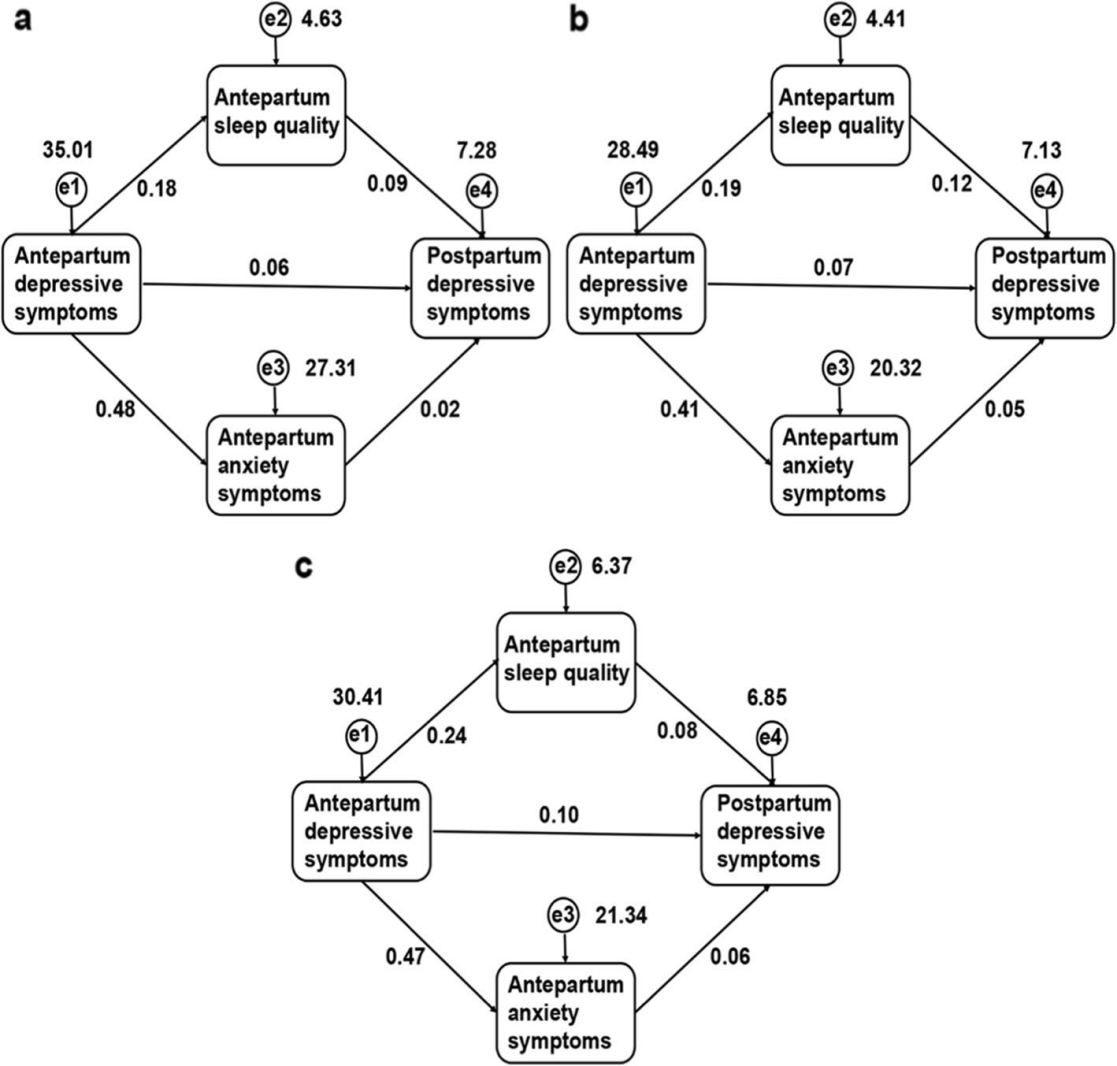
Mediation models for the relationship between antepartum depressive symptoms and postpartum depressive symptoms. **a** Mediation model of antepartum sleep quality and antepartum anxiety symptoms on the relationship between antepartum depressive symptoms and postpartum depressive symptoms in the first trimester. **b** Mediation model of antepartum sleep quality and antepartum anxiety symptoms on the relationship between antepartum depressive symptoms and postpartum depressive symptoms in the second trimester. **c** Mediation model of antepartum sleep quality and antepartum anxiety symptoms on the relationship between antepartum depressive symptoms and postpartum depressive symptoms in the third trimester

Figure 2 displayed the mediation analyses among antenatal sleep quality, mental health and postpartum depressive symptoms. The CES-D score was used as the observed variable. The PSQI and SAS scores were the intermediate variables, and the EPDS score was the dependent variable. Throughout the pregnancy, both poor sleep quality and anxiety symptoms mediated the effect of antepartum depressive symptoms on postpartum depressive symptoms. In the first trimester model, χ^2^/df = 0.04, GFI = 1.000, CFI = 1.000, TLI = 1.009, NFI = 1.000, RFI = 1.000, and RMSEA = 0.000. In the second trimester model, χ^2^/df = 1.963, GFI = 0.999, CFI = 0.999, TLI = 0.991, NFI = 0.997, RFI = 0.982, and RMSEA = 0.027. In the third trimester model, χ^2^/df = 1.967, GFI = 0.999, CFI = 0.999, TLI = 0.993, NFI = 0.998, RFI = 0.985, and RMSEA = 0.027. These models were well fitted. The results showed that the total and direct effects of antepartum depressive symptoms on postpartum depressive symptoms were of statistical significances across trimesters. The direct effects of antepartum sleep quality and antepartum anxiety symptoms on postpartum depressive were also statistically significant except for the effect of antepartum anxiety symptoms in the first trimester on postpartum depressive symptoms. The indirect effects of antepartum poor sleep quality and antepartum anxiety symptoms on the relationship of antepartum depressive symptoms and postpartum depressive symptoms showed statistical significances as well. In the first trimester, the effect of poor sleep quality and anxiety symptoms antenatally explained 32.14% of the relationship between antepartum depressive symptoms and postpartum depressive symptoms (*P* = 0.002). In the second and third trimesters, antepartum poor sleep quality and antepartum anxiety symptoms accounted for 39.25% and 31.25%, respectively, of the relationship between antepartum depressive symptoms and postpartum depressive symptoms (*P* = 0.001 and *P* = 0.001, respectively) (Table 3).

**Table 3:** Mediation analysis results for the relationship between antepartum depressive symptoms and postpartum depressive symptoms

	**Total effect**		**Direct effect**		**Indirect effect**		**Percentage mediated**
	**Effect size (95% CI)**	***P* value**	**Effect size (95% CI)**	***P* value**	**Effect size (95% CI)**	***P* value**	
**1**^**st**^** trimester**							
CES-D	0.084 (0.056, 0.112)	0.001	0.057 (0.026, 0.090)	0.001	0.027 (0.010, 0.044)	0.002	32.14%
PSQI	0.090 (0.029, 0.153)	0.003	0.090 (0.029, 0.153)	0.003			
SAS	0.022 (-0.005, 0.050)	0.120	0.022 (-0.005, 0.050)	0.120			
**2**^**nd**^** trimester**							
CES-D	0.107 (0.076, 0.140)	0.001	0.066 (0.032, 0.105)	0.001	0.042 (0.023, 0.062)	0.001	39.25%
PSQI	0.115 (0.044, 0.183)	0.002	0.115 (0.044, 0.183)	0.002			
SAS	0.048 (0.015, 0.084)	0.008	0.048 (0.015, 0.084)	0.008			
**3**^**rd**^** trimester**							
CES-D	0.144 (0.118, 0.171)	0.001	0.099 (0.064, 0.131)	0.001	0.045 (0.025, 0.067)	0.001	31.25%
PSQI	0.079 (0.021, 0.141)	0.009	0.079 (0.021, 0.141)	0.009			
SAS	0.057 (0.024, 0.086)	0.001	0.057 (0.024, 0.086)	0.001			

*CES-D* Center for Epidemiologic Studies Depression Scale, *PSQI* Pittsburgh Sleep Quality Index, *SAS* Self-Rating Anxiety Scale, *CI* Confidential interval

## Discussion

In this longitudinal cohort study, we found women who experienced poor sleep quality and mental health problems (depressive/anxiety/stress symptoms) antenatally were at higher risk of postpartum depressive symptoms. The sleep quality, depression and anxiety scores presented U-shaped curves across the trimesters while the perceived stress scores followed a descending trend in both groups. Importantly, we found that the impact of antepartum depressive symptoms on the development of postpartum depressive symptoms was mediated by poor sleep quality and anxiety symptoms across the whole pregnancy. These findings provide good insight in areas to target preventative strategies within the Chinese culture.

In this prospective longitudinal study, we followed up the participants from early pregnancy into the postpartum period and depicted sleep quality and mental health trajectories across the three trimesters. Interestingly, the curves for the sleep quality, depression and anxiety scores were alike, all presenting U-like shapes, suggesting a strong interconnection among these variables across time. This finding is supported by previous research demonstrating poor sleep quality and emotional distress are comorbid and strongly intertwined [33, 34]. However, maternal perceived stress scores were not U-shaped but rather represented a decrease trend from the first to third trimester, especially among those who did not develop postpartum depressive symptoms. These trajectory findings are consistent with previous research showing that maternal depression and anxiety rates were elevated in the first and third trimesters with a decline in the second trimester [35]. The elevation in early pregnancy may be due to an unwanted pregnancy, fear of a miscarriage, or morning sickness [36]. The secondary peak in late pregnancy may be attributable to increased physical discomfort and worry about the impending labor process and future parenthood [37]. The decreased perceived stress scores across time found in our study maybe due to gradual adjustment and growing confidence as the pregnancy progresses among these pregnant women. An understanding of how sleep quality changes during pregnancy is still lacking. Our result on the trajectory of sleep quality across pregnancy is similar with the results reported by small-sample studies of American and Canadian women [38, 39]. These studies illustrated that race and emotional problems influenced the trajectory the sleep, and thus the relationship between sleep quality and mental status in the perinatal period is worth investigation in Chinese population. Even though previous studies have investigated different trajectories of mothers’ depressive, anxiety and stress symptoms as well as sleep quality during pregnancy [40–42], it is of great value to further explore the associations between patterns of mental status and sleep quality in pregnancy and postpartum depression.

We found that sleep quality and mental health status in each trimester were associated with the occurrence of postpartum depressive symptoms after adjusting for the confounding factors. Given the effect of trimester, we used mixed-effects models to detect differences of the sleep quality, depression, anxiety and perceived stress scores across trimesters between the two groups, and the results also showed significant differences in these scores by group. Although the effect of trimester × group on sleep quality and depression/anxiety symptoms did not present significant differences, the effect of trimester × group on perceived stress did. Thus, the gaps in sleep quality and depression/anxiety symptoms between the two groups remained stable, while the gap in stress level became increasingly obvious as pregnancy progressed. These results suggest that early identification and the provision of secondary preventive strategies should be formulated as early as possible to avoid the occurrence of postpartum depression. Recently in a small sample of women, a significant trimester-by-race/ethnicity interaction was observed for anxiety rather than depression [43], suggesting the severity of mental distress may differ in trimesters among different populations. In the future, the mental health/sleep quality-by-trimester interaction for postpartum depression needs further investigation.

Though preceding studies have shown that antenatal sleep and mental health are strongly interrelated and are both potent determinants of postpartum depression, the causality of the relationship between disturbed sleep and mental health is still not clear [34, 44]. Some studies have suggested a bidirectional relationship between them [45]. To further clarify the association between sleep quality and mental health, we established mediation models for each trimester to explain the distinct interactions among antenatal sleep quality and mental health towards the development of postpartum depressive symptoms. In each trimester, the antenatal depressive effect on postpartum depressive symptoms is mediated by poor sleep quality and anxiety symptoms, and the percentage of the effect explained by this mediation is outstanding in the second trimester. In the models, the direct effects of antepartum depression and anxiety on postpartum depressive symptoms increased across the trimesters, while the direct effect of antepartum sleep quality on postpartum depressive symptoms peaked in the second trimester. These findings provide us with detailed clues to better understand the relationship among antenatal sleep quality, mental health and postpartum depressive symptoms as well as enlighten us how to establish advisable strategies to prevent postpartum depression, suggesting that sleep and anxiety interventions antenatally can be taken into considerations. Recently, it was reported that peripartum depressive symptoms could be relieved by improved sleep quality [46]. However, another clinical trial evaluating sleep psychoeducational intervention among healthy pregnant women, found no beneficial effect on the development of depressive symptoms [47]. Further intervention research among women at higher risks of insomnia and/or depression is warranted. While perinatal depression is gaining wide attention and screening for peripartum depression is extensively recognized, little effort on screening for perinatal anxiety disorders is executed [48]. However, perinatal anxiety symptoms are also quite common, and can also cause poor obstetric and postnatal outcomes [49]. In the study, antenatal depressive and anxiety symptoms can both directly impact postnatal depressive symptoms, and antenatal depressive symptoms can indirectly impact postnatal depressive symptoms through antenatal anxiety symptoms, so it is of meaning to conduct antenatal depressive and anxiety screenings and provide suitable interventions for high-risk pregnant women.

In addition, we examined some frequently identified influencing factors for postpartum depression including age, parity, marital status, and socioeconomic status [18, 50]. In our study, we found no demographic characteristic was significantly associated with postpartum depressive symptoms. We also examined the effect of exercise status on postpartum depression. In the present study, we found that more frequency of exercise during the third trimester had a protective effect against postpartum depressive symptoms. To prevent depression in general, meta-analytic data suggests physical exercise may be an effective strategy [51]. An emerging body of research support the benefits of antenatal physical activity on maternal mood [52, 53]. In a meta-analysis of 12 studies completed by Poyatos-Léon et al., physical activity interventions delivered in pregnancy moderately decreased the risk of developing postpartum depressive symptoms [54]. Exercise interventions that started in the first trimester have been shown to reduce depressive symptoms in the third trimester and postnatally [55]. Accordingly, strategies to promote the continuation of physical activity throughout the pregnancy and into the third trimester should be explored and include modifying exercise activities and routines to accommodate fetal development and the physical changes of pregnancy.

Our study is a prospective longitudinal study with a relatively large sample size. The delineation of the change patterns of sleep quality and mental status associated with subsequent postpartum depressive symptoms adds robust evidence to the existing literature. Further, the mediation analysis, in investigating the mediating effect of antenatal sleep quality and anxiety symptoms on the relationship between antepartum depressive symptoms and postpartum depressive symptoms, is a novel contribution of the study. However, the assessments of antenatal sleep quality, mental health and postpartum depressive symptoms were derived from self-reported questionnaires, which may be susceptible to bias.

## Conclusions

In summary, this study suggests that postpartum depressive symptoms are associated with antenatal sleep quality and psychological health. Sleep quality and psychological status vary during pregnancy. Early screening and interventions should be implemented to prevent the occurrence of postpartum depression.

## Supplementary Information

**Additional file 1: Supplementary Table 1. **Comparison of psychological health of the participants (PSQI≤5 vs. PSQI>5 in the 1^st^ trimester) in the perinatal period. **Supplementary Table 2.** Characteristics of the pregnant women and neonates between the two groups in the cohort study. **Supplementary Table 3.** Antenatal sleep quality and mental distress predict the risk of postpartum depressive symptoms.

## Data Availability

The datasets used and/or analyzed during the current study available from the corresponding author on reasonable request.

## Abbreviations

CNBC: China National Birth Cohort
PSQI: Pittsburgh Sleep Quality Index
CES-D: Center for Epidemiologic Studies Depression Scale
EPDS: Edinburgh Postnatal Depression Scale
SAS: Zung Self-Rating Anxiety Scale
PSS: Perceived Stress Scale
BMI: Body mass index
GWG: Gestational weight gain
IQR: Interquartile range
GDM: Gestational diabetes mellitus
χ2/df: Chi-square/degrees of freedom
GFI: Goodness of fit index
CFI: Comparative fit index
TLI: Tucker-Lewis index
NFI: Normed fit index
RFI: Relative fit index
RMSEA: Root mean square error of approximation
