# Supplementary material for: Antepartum sleep quality, mental status, and postpartum depressive symptoms: a mediation analysis
Source: BMC Psychiatry. 2022 Aug 2;22:521. doi: 10.1186/s12888-022-04164-y (PMC9344627; doi:10.1186/s12888-022-04164-y)
Supplement: Supplementary file 1 — Additional file 1: Supplementary Table 1. Comparison of psychological health of the participants (PSQI≤5 vs. PSQI>5 in the 1st trimester) in the perinatal period. Supplementary Table 2. Characteristics of the pregnant women and neonates between the two groups in the cohort study. Supplementary Table 3. Antenatal sleep quality and mental distress predict the risk of postpartum depressive symptoms. [file 12888_2022_4164_MOESM1_ESM.docx]

| **Supplementary Table1 Comparison of psychological health of the participants (PSQI≤5 vs. PSQI>5 in the 1^st^ trimester) in the perinatal period.** | | | |
| --- | --- | --- | --- |
| **Measurements** | **PSQI≤5 (n=787) No. (%)** | **PSQI>5 (n=514) No. (%)** | ***P* value** |
| **Psychological health in the 1^st^ trimester** | | | |
| CES-D |  |  |  |
| Total score, Median (IQR) | 16 (13-20) | 21 (17-25) | <0.001 |
| <16 | 355 (45.11) | 78 (15.18) | <0.001 |
| ≥16 | 432 (54.89) | 436 (84.82) |  |
| SAS |  |  |  |
| Total score, Median (IQR) | 45 (41-47) | 47 (42-51) | <0.001 |
| <50 | 661 (83.99) | 346 (67.32) | <0.001 |
| ≥50 | 126 (16.01) | 168 (32.68) |  |
| PSS, Median (IQR) | 19 (16-21) | 20 (18-21) | <0.001 |
| **Psychological health in the 2^nd^ trimester** | | | |
| CES-D |  |  |  |
| Total score, Median (IQR) | 14 (12-18) | 17 (13-21) | <0.001 |
| <16 | 501 (63.66) | 223 (43.39) | <0.001 |
| ≥16 | 286 (36.34) | 291 (56.61) |  |
| SAS |  |  |  |
| Total score, Median (IQR) | 43 (41-47) | 45 (42-48) | <0.001 |
| <50 | 709 (90.09) | 412 (80.16) | <0.001 |
| ≥50 | 78 (9.91) | 102 (19.84) |  |
| PSS, Median (IQR) | 18 (16-20) | 19 (17-21) | <0.001 |
| **Psychological health in the 3^rd^ trimester** | | | |
| CES-D |  |  |  |
| Total score, Median (IQR) | 14 (12-18) | 17 (13-22) | <0.001 |
| <16 | 490 (62.26) | 199 (38.72) | <0.001 |
| ≥16 | 297 (37.74) | 315 (61.28) |  |
| SAS |  |  |  |
| Total score, Median (IQR) | 45 (41-47) | 45 (42-48) | <0.001 |
| <50 | 679 (86.28) | 387 (75.29) | <0.001 |
| ≥50 | 108 (13.72) | 127 (24.71) |  |
| PSS, Median (IQR) | 18 (15-20) | 18 (16-21) | 0.004 |
| **Psychological health in 6 weeks postpartum** | | | |
| EPDS |  |  |  |
| Total score, Median (IQR) | 7 (5-9) | 8 (6-10) | <0.001 |
| <10 | 628 (79.80) | 356 (69.26) | <0.001 |
| ≥10 | 159 (20.20) | 158 (30.74) |  |
| PSQI: Pittsburgh Sleep Quality Index, CES-D: Center for Epidemiologic Studies Depression Scale, SAS: Self-Rating Anxiety Scale, PSS: Perceived Stress Scale, EPDS: Edinburgh Postnatal Depression Scale | | | |

| **Supplementary Table2 Characteristics of the pregnant women and neonates between the two groups in the cohort study.** | | | |
| --- | --- | --- | --- |
|  | **EPDS<10 (n=984) No. (%)** | **EPDS≥10 (n=317) No. (%)** | ***P* value** |
| **Parity** |  |  | 0.406 |
| Nulliparous | 636 (64.63) | 213 (67.19) |  |
| Multiparous | 348 (35.37) | 104 (32.81) |  |
| **Preterm birth** |  |  | 0.220 |
| No | 952 (96.75) | 302 (95.27) |  |
| Yes | 32 (3.25) | 15 (4.73) |  |
| **Delivery mode** |  |  | 0.608 |
| Vaginal delivery | 555 (56.40) | 184 (58.04) |  |
| Cesarean delivery | 429 (43.60) | 133 (41.96) |  |
| **Newborn weight, Median (IQR) (kg)** | 3.36 (3.09-3.61) | 3.32 (3.02-3.60) | 0.195 |
| **Newborn length, Median (IQR) (cm)** | 50.00 (50.00-50.00) | 50.00 (50.00-50.00) | 0.115 |
| **Twins** |  |  | 1.000 |
| No | 977 (99.29) | 315 (99.37) |  |
| Yes | 7 (0.71) | 2 (0.63) |  |
| **Sex** |  |  | 0.754 |
| Male | 476 (48.03) | 150 (47.02) |  |
| Female | 515 (51.97) | 169 (52.98) |  |
| **Gestational diabetes mellitus** |  |  | 0.024 |
| No | 835 (84.86) | 285 (89.91) |  |
| Yes | 149 (15.14) | 32 (10.09) |  |
| **Hypertensive disorders of pregnancy** | | | 0.343 |
| No | 906 (92.07) | 297 (93.69) |  |
| Yes | 78 (7.93) | 20 (6.31) |  |
| **Placenta previa** |  |  | 0.202 |
| No | 972 (98.78) | 310 (97.79) |  |
| Yes | 12 (1.22) | 7 (2.21) |  |
| **Placental abruption** |  |  | 1.000 |
| No | 977 (99.29) | 315 (99.37) |  |
| Yes | 7 (0.71) | 2 (0.63) |  |
| **Intrahepatic cholestasis** |  |  | 0.200 |
| No | 965 (98.07) | 307 (96.85) |  |
| Yes | 19 (1.93) | 10 (3.15) |  |
| **Intrauterine distress** |  |  | 0.326 |
| No | 771 (78.35) | 240 (75.71) |  |
| Yes | 213 (21.65) | 77 (24.29) |  |
| **Fetal growth restriction** |  |  | 0.589 |
| No | 976 (99.19) | 316 (99.68) |  |
| Yes | 8 (0.81) | 1 (0.32) |  |
| **Hyperthyroidism** |  |  | 1.000 |
| No | 980 (99.59) | 316 (99.68) |  |
| Yes | 4 (0.41) | 1 (0.32) |  |
| **Hypothyroidism** |  |  | 0.981 |
| No | 934 (94.92) | 301 (94.95) |  |
| Yes | 50 (5.08) | 16 (5.05) |  |
| **Meconium-stained amniotic fluid** |  |  | 0.869 |
| No | 808 (82.11) | 259 (81.70) |  |
| Yes | 176 (17.89) | 58 (18.30) |  |
| **Anemia in pregnancy** |  |  | 0.218 |
| No | 822 (83.54) | 274 (86.44) |  |
| Yes | 162 (16.46) | 43 (13.56) |  |
| EPDS: Edinburgh Postnatal Depression Scale | | | |
|  |  |  |  |

| **Supplementary Table3** **Antenatal sleep quality and mental distress predict the risk of postpartum depressive symptoms.** | | | |
| --- | --- | --- | --- |
|  | **PSQI [OR (95% CI)]**  **（PSQI>5）** | **CES-D [OR (95% CI)]**  **（CES-D≥16）** | **SAS [OR (95% CI)]**  **（SAS≥50）** |
| **1^st^ trimester** |  |  |  |
| Crude | 1.75 (1.36, 2.26) | 2.49 (1.83, 3.38) | 1.88 (1.41, 2.49) |
| Adjusted^*^ | 1.80 (1.39, 2.33) | 2.44 (1.80, 3.32) | 1.92 (1.44, 2.55) |
| **2^nd^ trimester** |  |  |  |
| Crude | 1.74 (1.33, 2.28) | 2.56 (1.98, 3.33) | 2.52 (1.82, 3.51) |
| Adjusted^*^ | 1.76 (1.34, 2.31) | 2.56 (1.97, 3.32) | 2.52 (1.81, 3.51) |
| **3^rd^ trimester** |  |  |  |
| Crude | 1.58 (1.22, 2.04) | 3.06 (2.34, 4.00) | 2.52 (1.87, 3.40) |
| Adjusted^#^ | 1.53 (1.18, 1.98) | 3.01 (2.30, 3.94) | 2.42 (1.78, 3.27) |
| PSQI: Pittsburgh Sleep Quality Index, CES-D: Center for Epidemiologic Studies Depression Scale, SAS: Self-Rating Anxiety Scale, OR: odds ratio, CI: confidential interval  The PSQI, CES-D and SAS scores are treated as categorical variables. | | | |
| *ORs and 95% CIs were adjusted for age, employment status, education level and exercise status. | | | |
| #ORs and 95% CIs were adjusted for age, employment status, education level, exercise status and gestational diabetes mellitus. | | | |
